# A Conceptual Design of Smart Management System for Flooding Disaster

**DOI:** 10.3390/ijerph18168632

**Published:** 2021-08-16

**Authors:** Thaer Ibrahim, Alok Mishra

**Affiliations:** 1 Department of Technical Affairs and Studies, Iraqi Federal Board of Supreme Audit, Baghdad 10002, Iraq; thairking78@gmail.com; 2 Faculty of Logistics, Molde University College-Specialized University in Logistics, 6410 Molde, Norway; 3 Department of Software Engineering, Atilim University, Ankara 06830, Turkey

**Keywords:** information and communication technology, smart disaster management system, Internet of Things, flood, disaster

## Abstract

Disasters pose a real threat to the lives and property of citizens; therefore, it is necessary to reduce their impact to the minimum possible. In order to achieve this goal, a framework for enhancing the current disaster management system was proposed, called the smart disaster management system. The smart aspect of this system is due to the application of the principles of information and communication technology, especially the Internet of Things. All participants and activities of the proposed system were clarified by preparing a conceptual design by using The Unified Modeling Language diagrams. This effort was made to overcome the lack of citizens’ readiness towards the use of information and communication technology as well as increase their readiness towards disasters. This study aims to develop conceptual design that can facilitate in development of smart management system for flooding disaster. This will assist in the design process of the Internet of Things systems in this regard.

## 1. Introduction

A disaster is an abnormal event that local capabilities cannot control and requires national or international assistance [1]. Disasters can be classified into two types: natural disaster and man-made disaster [2]. Flood is one of the natural disasters caused by the high level of fresh water or salt water [3]. Natural disasters have a huge negative impact on people’s lives and property by killing, injuring, and displacing people. Floods were among the most common natural disasters in the last decade, reaching about 50% of the natural disasters rate [4]. Due to damages caused by disasters, many private sectors and public organizations have tried to develop policies, frameworks, and systems for coping with future disasters by addressing these disasters in a scientific and deliberate manner [5]. Additionally, the necessary plans should be prepared in advance before the disaster strikes; this procedure is called a disaster management system (DMS) [6]. Natural disasters can be defined as a group of violent natural activities that occur suddenly in populated areas, causing heavy casualties and preventing people from taking precautions to mitigate their destructive effects [7]. One of the most important responsibilities of any government is to protect its citizens and national resources from any threats, especially natural disasters. In addition, one of the main services provided by the government to its citizens is security, which is defined as preserving the life and property of citizens from damage, whether due to humans or the surrounding environment [8].

The smart disaster management system (SDMS) is a DMS modified by the addition of information and communication technology (ICT) [9]. This system takes advantage of the facilities provided by ICT to accelerate the delivery of information and reduce the rate of error in decision-making in the event of a disaster. The intelligence of this system comes from its ability to both collect information related to the disaster and make decisions in real time [10]. The motivation behind the design of an SDMS is to increase the readiness of people to use ICT in a correct and efficient manner. In addition, it aims to increase the citizens’ reliability on E-government applications by enhancing the utility of these applications. Another important aim is increasing the citizens’ readiness for disaster management through facilitating the ways of communication between people and the SDMS.

Flooding is the failure of the waterway to absorb rainwater to exceed its natural course and inundate the surrounding areas [11]. Flood can be addressed as one of these natural disasters. The continuous suffering of mankind from the impact of flood disaster over time was the main motivation for the development of this research. The International Federation of Red Cross and Red Crescent Societies (IFRCS) estimated that between 2008 and 2017 about 8.6 million people were displaced because of disasters (flooding). Over the last decade of the twentieth century, floods have influenced some 1.5 billion individuals. Around 200 million people worldwide live-in coastal areas prone to flooding [12].

Floods more often than not are the result of extreme and consistent precipitation that surpasses the absorptive capacity of the soil and the draining capacity of streams and rivers, especially in coastal regions. Thunderstorms, tornadoes, tropical cyclones, monsoons, melting ice and dams could cause flooding as well. The foremost types of floods are flash flooding, snow-melting surges, coastal flooding, and waterway flooding. Sudden flash surges are the most perilous since they happen unexpectedly, particularly at night [13].

Increased population density, fast and poorly planned urbanization, such as destruction of timberlands and natural confinement zones that prevent destructive floods, and climate alteration are all major factors of floods. As these processes are increasing, more individuals will be exposed to future floods. Dissolving ice sheets and rising ocean levels will cause flooding in areas that had not been exposed to surge hazards. Developing countries are at the greatest risk, and although Asia remains the worst continent hit by surges, Africa and Latin America have been affected more frequently in recent years. It should be emphasized that wealthy nations are not safe from the threats of flooding.

Flood disaster is one of the most frequent natural disasters that occur all around the globe [14]. Based on a research conducted by Institute of Environmental Studies, more than 60% of world cities be vulnerable to flooding in next 30 years due to climate changes effects and it will also lead to sea-level rise [15]. In 2014, natural disasters caused 7700 fatalities and losses of USD110 billion worldwide [16]. Flood occurring is the common disaster among all disaster [17]. In Australia, flooding accounts for an average of USD377 million in damages annually [18]. Applying and integrating sensory information in disaster management has gained attention in recent years as an efficient solution for providing live disaster information [19,20]. The application of sensors to measure the hydrological data and transmission over network has become common in many countries; presently, the Internet of Things (IoT) has been focused to improve disaster management on flood [14]. Therefore, this study pursues further advances in this regard.

## 2. Background and Related Work

The main aim of this study is to provide a conceptual design to advance in the development of a smart management system for flooding disaster. According to the World Meteorological Organization (WMO), flooding remains the third biggest disaster in the world [21]. Presently, IoT has become one of the technologies that has been used as a study to improve the area of disaster management focusing on flood [14]. They provided a review on application of IoT for flood data management and proposed an IoT architecture in this regard. IoT can be used to monitor the environment such as water level, flow rate, rainfall, and many other things. Thus, there are many early warning disaster systems integrated with IoT [22]. Inputs to flood forecasting system include many hydrological, meteorological, and geological data collected from an extensive network of monitoring stations. The data includes river level and river flow, rainfall and tide, and these data originate from various devices [23]. Based on the result of experiments conducted, the flood early warning system worked properly as observed by the accuracy of the prototype achieved, which has a 95.6% accuracy [22]. Rismayana et al. (2018) [24] proposed a prototype system of flooding early warning system using IoT and social media. Their system serves to measure the water level at any time and send data in real time to the server and communicates the warning message to all registered telegram contacts. Ji and Anwen [25] proposed an application framework of IoT in an emergency management system in China. Chen et al. (2013) [26] developed an IoT application for safe buildings, where this application focused on helping people escape from the buildings in case there is a fire or an earthquake. Mitra et al. (2016) [27] proposed an IoT and machine learning-based embedded system to predict the probability of floods in a river basin. An IoT-based water monitoring system that measures the water level was proposed by Perumal et al. (2015) [28]. Mane et al. (2015) [29] developed a flood forecasting system using data mining and a wireless sensor network. Du and Zhu (2012) [30] investigated the application of IoT technologies in monitoring, controlling real-time data, and accurate prediction of early warning for urban public safety emergency management.

Ghapar et al. (2013) [14] reviewed research works that utilize IoT for flood data management and proposed an IoT architecture for flood data management that can facilitate for IoT infrastructure implementation. Kumar et al. (2019) [31] explored the research challenges of designing an information framework that couples physical sensors with the social sensors to collect heterogeneous flooding disaster data, and then to fuse that data and generate actionable understandings. The goal was to improve the response preparedness of critical infrastructures, contributing to the goals of smart and connected communities [32]. Data from sensors can be used for developing early warning processes towards useful early warning for flood management. Prevailing experience indicates that several devices have been developed based on sensor data and integrated satellite such as TRMM rainfall, Radar sat SAR, and Namibia Flood Sensor Web [32]. Therefore, this study will facilitate in developing a conceptual design of (SDMS) for flood warning. The rest of the paper is structured as follows: Section 3 presents the literature review. Section 4 describes the methodology. Section 5 provides the discussion. Finally, Section 6 includes the conclusion, limitations, and future work.

## 3. Literature Review

In the field of flooding and aquatic monitoring. Table 1. below shows the recent studies along with the main themes of these studies:

Disaster management can be divided into three stages [39]:

### 3.1. Before Disaster

In this stage, the disaster management team has the following duties:

Take all the necessary preventive measures, both administrative and executive, to save the lives and property of citizens and minimize losses to the lowest possible rate.

Review all studies on disaster risk reduction and take into account all proposals referred to in these studies.

Stay alert in the event of a disaster to ensure prompt and effective action.

Raise awareness among the citizens and create a well-informed society in order to obtain their assistance in the event of disasters, thus reducing the damage to a minimum.

### 3.2. During Disaster

This is the most critical stage for the disaster management team because of the chaos caused by the disaster; therefore, the team has deferent responsibilities, such as [40]:

Provide decision-makers with real-time data about the disaster, people affected, and the physical damage.

Saving as many afflicted citizens as possible.

Coordinate with government institutions, civil society organizations, and citizens trained in first aid to maximize their efforts in rescuing citizens.

Act directly to rescue people injured or trapped under rubble.

### 3.3. After Disaster

At this stage, the disaster management team has the following duties:

Provide suitable shelters for citizens who have lost their homes.

Provide affected people with the necessary needs for daily life, such as food and medicine.

Provide material and moral compensation to citizens who suffered losses during the disaster.

## 4. Methodology

In order to enhance the (DMS), a conceptual design was developed by supposing a disaster scenario; this scenario must be visualized by:

- Defining the system requirements.
- Using a use case diagram approach to visualize the role of participants.
- Using a class diagram approach to visualize the system activities.

Therefore, a scenario will be prepared for each stage to explain the activities and responsibilities of each person within the system. The system stages are explained as follows:

1. Before Flood Disaster Stage

This stage is the pre-disaster stage and includes all the physical and psychological preparations needed in case a flood disaster strikes; the scenario of this stage is as follows:

- System requirements
  1. PCs, sensors, networks, data centers, database (DB), and Internet service.
  2. Smart devices containing Global Positioning System (GPS) software.
  3. The alarming system connected to AI software.
  4. The training center containing all training requirements to train citizens and disaster teams.
  5. Food, medicines, and equipment to be used during and after the disaster.
- People
  1. Disaster Management Team
    - Team leader: this person is responsible for declaring a state of emergency in the event of a disaster and has the authority to issue instructions to the disaster management and support teams as well as volunteers. This person is also responsible for the management of disaster from the moment of its occurrence until the last stage. Therefore, this person must have administrative authority, such as being a governor or the city mayor.
    - Team members: as members of the disaster management team, they are experienced in dealing with all types of disasters and have sufficient skills to train volunteers and control any emergency during a disaster. Members of the team must always be in a state of readiness for disasters.
  2. Supporting

Supporting staff are non-permanent members of the disaster management team, but when a disaster occurs, they are on alert to help the disaster management team. In addition, they must be skilled in dealing with emergencies. These are:

   • Police officers: police officers have an important role in the event of disasters, as they have experience in first aid and various other ways to deal with emergencies. In addition, they can protect important facilities from tampering and theft.
Firefighters: the main role of firefighters is to put out the fires caused by the disaster; they are also essential in rescuing people trapped in the rubble because of the collapse of buildings. They also have experience in first aid.
Paramedics: the primary duty of paramedics is to provide first aid to injured people and transfer serious cases to the hospital in the event of disasters.
Transporters: transporters evacuate people affected by the disaster to safe areas in order to preserve their lives. In addition, they must also be able to provide first aid during the disaster.
Volunteers: volunteers are civilians who have been trained by the disaster management teams on first aid, therefore, they are useful during disasters to save people and minimize casualties.

c. Use Case Diagram

**Figure 1:**
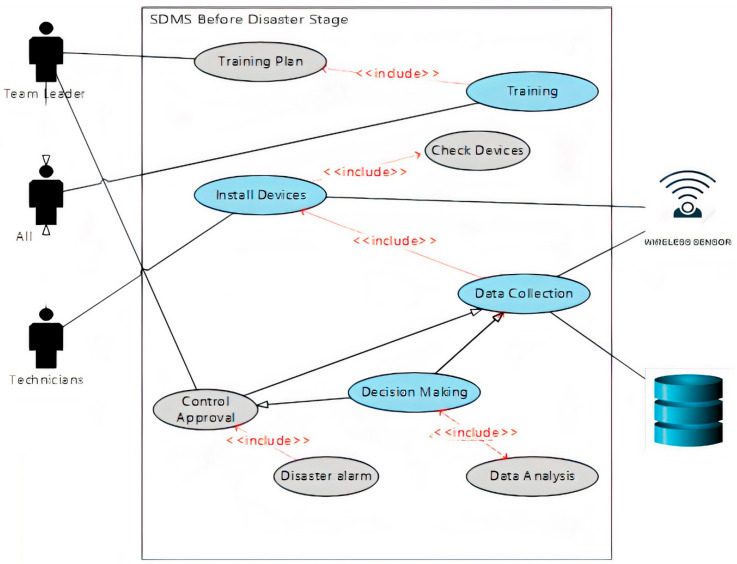
Use case diagram of before disaster stage [39].

Use case diagrams for the stage before disaster were used based on the advice of the Thesis Monitoring Committee and the recommendations of the system developers. The purpose of using these diagrams is to illustrate the roles of people involved, processes, and data flow within the system. Figure 1 shows a diagram for the stage before disaster in the proposed SDMS.

  1. The team leader is responsible for determining the training plan, its dates, the participants, and the necessary materials.
  2. All SDMS team, including the team leader, will participate in the training program in order to be ready to deal with the disaster.
  3. Technicians are responsible for installing the system hardware, connecting, and testing the network and conducting periodic checks on all parts to ensure it is working properly.
  4. Sensors start sending their data to the data center to check the monitored area for any abnormal situation.
  5. The data center will receive data and analyze it; if any abnormal pattern is detected, the system will compare the abnormal data with the historical data stored in the DB.
  6. If the system decides that there is danger, an alarm message will be sent to the team leader.
  7. The team leader will determine the risk ratio according to the data received from the data center.
  8. If the situation is controllable, the team leader will send the specialists to control the situation and send their report to the DB to be saved as historical data.
  9. If the level of risk is high, the team leader will declare an emergency and start the second phase of the system.

d. Flooding scenario
  1. The team leader sends an alarm message to the team members, supporting teams, and civilian volunteers, ordering them to start the flooding disaster plan.
  2. The first step in the flooding disaster plan is warning people of flood disaster by using all communication methods such as SMSs, social media, TV channels, and radios and encouraging them to go to the safe zone.
  3. Use radio-frequency identification (RFID) technology to detect the location of the elderly and the disabled in order to save them.
  4. Evacuate people and transport them to safe areas that have been prepared during the preparatory phase of the disaster.
  5. Cut off electricity, gas, and water sources to avoid any additional damage caused by them.
  6. Use rescue boats to save people at risk of drowning and recover bodies.
  7. Use water suction pumps to reduce the water pressure from the affected areas.
  8. Use loaders and lorries to make earth mounds that keep floodwaters in control.
  9. Use RFID to detect trapped people and save them.
  10. Provide first aid to people affected with minor injuries.
  11. Transport people who are seriously injured to the hospital.
  12. Keep the team leader informed about the latest updates of the disaster and the real situation on the ground.

2. During Flood Disaster Stage

This is the most dangerous and important stage for the SDMS. During this stage, events are handled in real time and the disaster management team must be adequately trained and equipped to deal with the situation. Successful management of this stage significantly reduces the loss of life and property.

The addition of ICTs to the DMS will significantly improve the performance of the system. GPS and IoT can be used to transmit information in real time and controlling many devices remotely can be an important factor in the success of disaster management and control at this stage. The scenario of this stage is as follows:

- Requirements
  1. Cell phones, smart devices, PCs, satellite channels, and internet service.
  2. Buses, ambulances, fire vehicles, police cars, fire fighting aircraft, rescue boats, lifeboats, loaders, and lorries.
  3. Sensors, RFIDs, wireless communication devices.
  4. Electric generators, water suction pumps, masks, respirators, and fire extinguishers.
- People
  1. Disaster management team (team leader, team members)
  2. Supporting (police officers, firefighters, pilots (to drive firefighting aircraft), divers (to search for sunken people), paramedics, transporters, volunteers).
- Responsibilities

**Figure 2:**
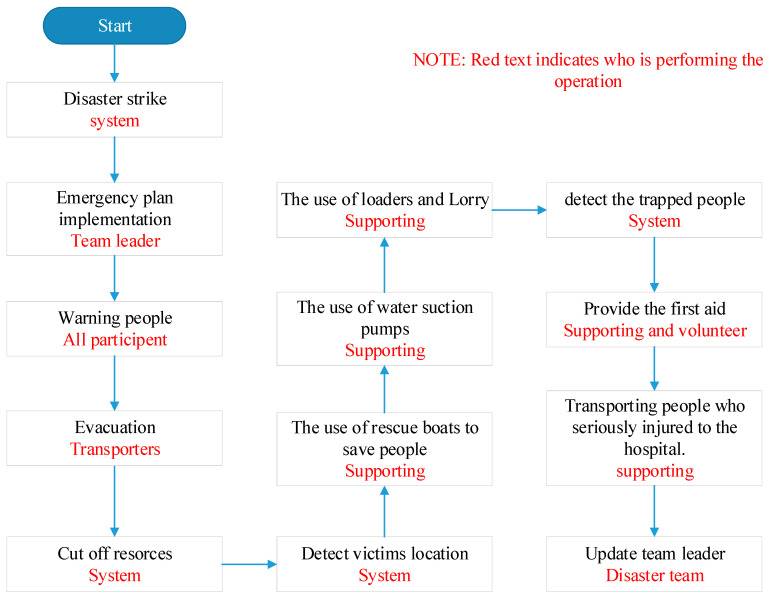
Flowchart of detailed activities of flooding during disaster stage [39].

In Figure 2, it can be seen that the SDMS system plays a central role, as it announces the occurrence of the disaster. It also manages all real-time data received in the database during the disaster. It also cuts off energy sources (electricity and fuel) automatically while the leader and members of the disaster management team are busy with their work. In addition, it locates victims and stranded people using GPS technology. All these properties are not found in the tradetional systems.

The figure also shows the role of the team leader (who is also considered a member of the team) as he/she supervises the team members and participates in all the activities carried out by the team in which the system has no role. The role of the team leader does not end, even after the disaster ends, as the team leader begins to manage the post-disaster phase and then prepares for upcoming disasters by training the team and preparing logistical support. As for the team members, their responsibilities vary according to their positions; some of them carry out the evacuation process, some drain the water, and some carry out the process of rescuing the injured and providing first aid to them, since in the disaster stage all citizens are considered members of the disaster management team.

Figure 2 shows the flowchart of main disaster stage activities. When the system receives real-time data that contains an emergency case or abnormal data related to the flooding, it directly informs the decision-makers or disaster team leader to confirm the disaster mode in order to start the disaster management procedures by mobilizing all team members as well as the volunteers. The SDMS will work in parallel with the disaster team depending on real-time data received from IoT sensors to cut off all power resources to avoid any fire or electricity danger.

The team will start the evacuation function with the help of the system to detect the victim’s location in order to provide the first aid to them or transport them to the hospital if their situation is so critical. The earlier works are carried out in conjunction with an attempt to reduce or control the water level to prevent the increase in the impact of the flood. Due to the advent of ICT applications, IoT sensors instruct controllers installed in electronic gates to open those gates in order to drain larger amounts of water to reduce the impact of flooding.

When the dangerous situation is over and the water returns to its normal level, the system will update the situation and move to the post-disaster stage. The team will begin repairing the damages, in addition to providing camps, medicine, and food for displaced people. When everything is returned back to normal, the team leader will announce the end of the disaster and return to standby mode.

d. Use case diagram of during the disaster stage

**Figure 3:**
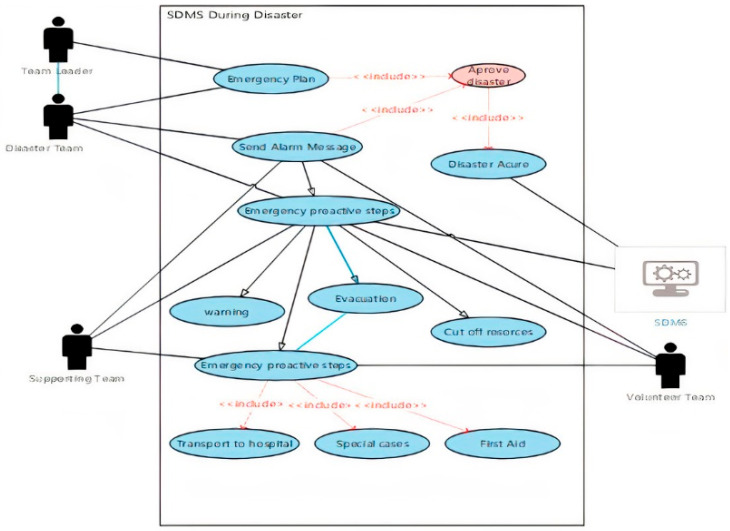
Use case diagram of during disaster stage [39].

The use case diagrams for the period during the disaster stage were designed according to the recommendations of the system developers. The purpose of using the use case diagram is to illustrate the roles of people, processes, and data flow within the system. This stage includes two diagrams: one for the main activities of this stage, and one for the special cases of flood disaster. Figure 3 below shows the use case diagram during the disaster stage of SDMS.

1. The team leader is responsible for announcing the disaster and approving the use of the emergency plan.
2. All SDMS teams will start warning people about the disaster.
3. The transporters who are part of supporting team will evacuate people to the safe zone.
4. The supporting team and the system will cut off the electricity, gas, and water to prevent additional damage.
5. The supporting team will deal with the special cases which will be explained in detail later.
6. The volunteer team will provide the first aid to injured people who are not in critical conditions.
7. Ambulances from the supporting team will transfer the critical cases to the hospital.

3. After Disaster Stage

This is the last stage in the SDMS, at the end of which the system will return to standby mode. During this stage, the disaster management team, supporting team, and civilian volunteers have different duties in different scenarios, which is as follows:

- Requirements
  1. Cell phones, smart devices, PCs, DB, Internet service, RFID, and statistics software.
  2. Tents, food, clean water, and medicine.
  3. Trucks, ambulances, police cars, generators, water treatment plants.
  4. Building materials.
- People
  1. Disaster management team (team leader, team members)
  2. Supporting (police officers, paramedics, statistics experts, technicians, construction workers, transporters)
  3. Volunteers
- Use case diagram of after disaster stage

**Figure 4:**
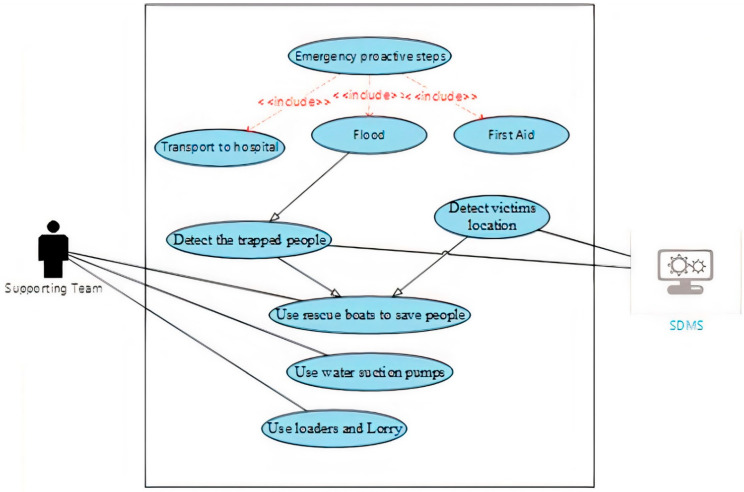
Use case diagram of during flood disaster stage [39].

The purpose of using such diagrams is to illustrate the roles of people, processes, and data flow within the system. Figure 4 below shows the use case diagram for the after-disaster stage.

  1. Statisticians as a support team with the help of SDMS will conduct statistical analyses to determine the number of surviving, injured, missing, and dead people, in addition to identifying material losses and damage to infrastructure.
  2. Based on the statistical report, the support team and volunteers will provide the survivors with the necessary food, drink, and medicine, as well as suitable places for living.
  3. Construction workers as a support team with the help of volunteers will remove the debris caused by the disaster.
  4. As a support team, construction workers will rehabilitate infrastructure and housing in the disaster-affected area.
  5. Technicians as a support team will rehabilitate the SDMS and its associated equipment with a thorough inspection of all parts of the system to make sure they are working properly.
  6. After the completion of the rehabilitation process, the team leader assesses the performance of the risk management team, supporters, and volunteers. He will analyze the speed of response and the way to better deal with the disaster. A copy of this report will be sent to the database to be saved as historical data training for the future.
  7. The team leader announces the end of the disaster and orders the team to return to standby mode.

**Figure 5:**
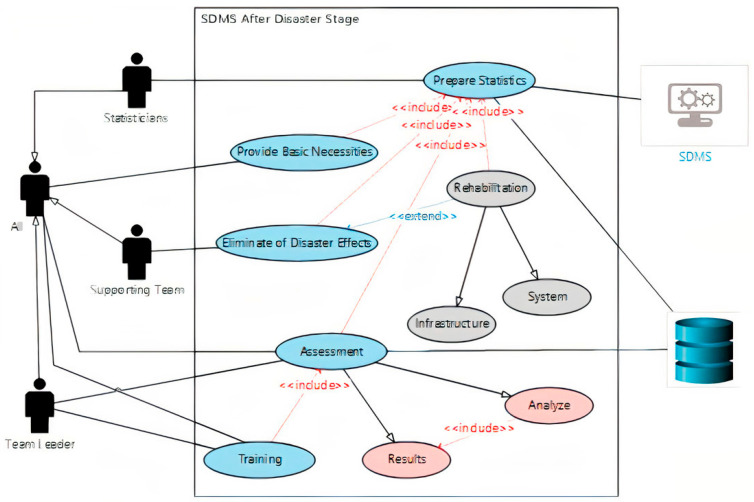
Use case diagram of after flood disaster stage [39].

Figure 5 below shows the activities and the responsibilities of the disaster team in the after flood disaster stage, represented in a Use case diagram. This figure also shows the important role of the SDMS and the DB in this stage.

## 5. Discussion

Natural disasters lead to geographic changes in the lives of people [41]. The goal of a DMS is to minimize the impact of a disaster [42]. In a similar manner, the United Nations Office for Disaster Risk Reduction (UNDDR) has defined disaster risk management (DRM) as “the process of using directives, organizations, and capacities to implement strategies policies, and improve capacities in order to decrease the impact of disasters” [43]. This system contains four facets: mitigation, preparedness, response, and recovery. All these facets are wholly dependent on IT [44]. The weakness of the existing DMSs is related to technical along with administrative vulnerabilities. This issue was argued by Nowell et al. [45], wherein they stressed that the success of the DMS relies on a change in the administrative structure of this system, making all units of the system linked to one administration and receiving orders from only one leader, in addition to linking all units of the system with each other by ICT.

Increasing the readiness of citizens towards disasters as well as increasing their ICT knowledge will contribute in reducing the effect of these disasters. The researcher agrees with this reflection, and it will be approved after the implementation of the SDMS. One of the most prominent challenges facing the implementation of the DMS is the high cost of the components of this system; therefore, most researchers in this field rely on conceptual design when trying to apply new ideas to this system [46]. For all the previous reasons, a conceptual design for an enhanced DMS called SDMS was proposed. Unified modelling language (UML) is used to describe the system structure and behavior. The interaction between different actors in the system is specified with use cases as part of UML diagram [47]. Interlinking every department through IoT allows to reduce the damages of disaster. Sensor networks, IoT, and embedded system structures can be used for the smart networks for emergency control management [33]. A metamodel-based knowledge sharing system for disaster management based on UML was proposed by Othman, and Beydoun [48]. They deploy the transformations specified in the meta-modelling framework of MOF, a standard for software metamodeling offered by OMG [49]. MOF defines a common way for capturing the diversity of modelling standards and interchange constructs that are used in model-driven software engineering. Othman and Beydoun [48] further argued that it provides a framework for defining modelling languages or information models for metadata. It uses an object-modelling framework that is essentially a subset of the UML core [50]. The application of ICT and IoT supports E-government services to facilitate in predicting and altering the consumers in order to reduce the damage caused by natural disasters and pollution [51]. Goniewicz and Burkle [52] developed an IT system for Poland’s Protection Against Extreme Hazards (ISOK); this system works to reduce the damages of physical and moral disasters to citizens by developing a crisis and risk management mechanism. In a similar way, another important system was implemented in Poland called the Sat4Envi project; this system works to gather, share, and promote for the environment by analyzing satellite images and obtain data related to disasters in order to forecast disasters in study by Goniewicz et al. [53].

In this research, a framework for enhancing the current DMS called SDMS is proposed. Here, the smart aspect of this system is due to the application of the principles of ICT, IoT technologies, and UML applications. This effort was made as part of doctoral study to overcome the lack of citizens’ readiness towards the application of ICT as well as an increase in their readiness towards handling such disasters.

## 6. Conclusions

The globe is exposed to various types of disasters; some of these disasters are natural and some are man-made. These disasters result in a substantial loss of life and property. Over the years, researchers have tried to control these disasters and reduce their losses. This research discussed the possibility of using (ICT), especially E-government applications, and the IoT in disaster management. The success of flood disaster management depends mainly on how well flood-related data can be collected, managed, and utilized. Due to this account, the application of IoT to facilitate flood data management is seen as a step in the right direction.

The concluding part of this research is proposing an enhanced DMS called SDMS in order to increase the readiness of citizens by applying recent ICT technologies such as IOT and AI to handle disasters. Where a conceptual design for the SDMS is prepared, this conceptual design contains a scenario for each stage of the disaster management system’s stages (before disaster, during disaster, and after disaster). UML diagrams are prepared for each stage to illustrate the participants, activities, and position of each person within the system. This will help in developing a conceptual design that can facilitate in the development of a smart management system for flooding disaster. This will also assist in the design process of the IoT systems in this regard.

In this conceptual design, the researchers tried to provide a general vision for the system developers, programmers, software engineers, and designers to work on system details, each according to their specific requirements (context) in order to stimulate their creativity. Therefore, this study did not address the implementation of such systems, for instance: specific type of devices, operating systems, databases, etc.; this may represent a kind of limitation for this research study. As future research directions, an integrated information system approach to the early warning of floods based on a geographical information system (GIS) and remote sensing could be designed and developed. Additionally, the implementation issue of such systems could be conducted as a case study with support from government agencies and related organizations.

This research did not receive any specific grant from funding agencies in the public, commercial, or not-for-profit sectors.

## Figures and Tables

**Table 1:** Recent studies with their contribution.

Wellington J, et al. (2017) [33]	Observed a lack of focus on the technical aspects of IoT technology. Argued that sensor networks, IoT, and embedded system structures can be used for the smart networks for emergency handling.
Rafi et al. (2018) [34]	Concluded an effective response to a disaster requires fast flow of information and integrated response activities; thus, computing technology can be helpful in this regard.
Shalini, et al. (2016) [35]	Designed a system to measure the level of water in the river using special sensors to measure water levels as the distance from the bottom of the river and send the data to the monitoring center using Wi-Fi technology, the information sent via smartphones using GSM technology to the decision-makers.
Organization of American States Disasters (OAS) (1990) [36]	Defined the (DMS) to harness the full potential of governmental and non-governmental institutions in the event of a disaster in order to minimize the damage caused by the latter.
K. Yao (2015) [37]	Provided another definition to the DMS, which is the system that is used to manage disaster-related data by using information technology (IT), which combines geographical information with administration information to facilitate access to and use of these data in all disaster stages.
Eraslan et al. (2004) [38]	Tried to change the administrative structure of the DMS by proposing the use of the communications side of (IoT) to connect all parts of the system with each other and make them work as one integrated unit. Then, they suggested the establishment of a central control unit that communicate with all other units of DMS using IoT.

## References

[1] Pascaline W., Rowena H. Economic Losses, Poverty & Disaster 1998–2017.

[2] EM-DAT General Classification. https://www.emdat.be/classification.

[3] Haraguchi M., Lall U. (2015). Flood risks and impacts: A case study of Thailand’s floods in 2011 and research questions for supply chain decision making. Int. J. Disaster Risk Reduct..

[4] Ritchie H., Roser M. Natural Disaster. https://ourworldindata.org/natural-disasters.

[5] Norio O., Ye T., Kajitani Y., Shi P., Tatano H. (2012). The 2011 eastern Japan great earthquake disaster: Overview and comments. Int. J. Disaster Risk Sci..

[6] Sakhardande P., Hanagal S., Kulkarni S. Design of disaster management system using IoT based interconnected network with smart city monitoring. Proceedings of the International Conference on Internet of Things and Applications (IOTA).

[7] Segal K., Jong J., Halberstadt J. (2018). The fusing power of natural disasters: An experimental study. Self Identity.

[8] Anthopoulos L.G., Reddick C.G. (2016). Understanding Electronic Government Research and Smart City: A Framework and Empirical Evidence. Inf. Polity.

[9] Alazawi Z., Altowaijri S., Mehmood R., Abdljabar M.B. Intelligent disaster management system based on cloud-enabled vehicular networks. Proceedings of the 11th International Conference on ITS Telecommunications (ITST).

[10] Alazawi Z., Alani O., Abdljabar M.B., Altowaijri S., Mehmood R. A smart disaster management system for future cities. Proceedings of the 2014 ACM International Workshop on Wireless and Mobile Technologies for Smart Cities.

[11] Fretas D., Battat W. (2019). The Impact of Flood Risk on Urban Areas.

[12] International Federation of Red Cross and Red Crescent Societies (IFRCRCS) (2018). World Disaster Report.

[13] Chan N.W. (2012). Impacts of disasters and disasters risk management in Malaysia: The case of floods. Economic and Welfare Impacts of Disasters in East Asia and Policy Responses.

[14] Ghapar A., Yussof S., Bakar A.A. (2018). Internet of Things (IoT) Architecture for Flood Data Management. Int. J. Future Gener. Commun. Netw..

[15] Ward P.J., Pauw W.P., Van Buuren M.W., Marfai M.A. (2013). Governance of flood risk management in a time of climate change: The cases of Jakarta and Rotterdam. Environ. Politics.

[16] Munich R. Topics Annual Review: Natural Disasters 2014. Mnchener Rckversicherungs-Gesellschaf. http://www.munichre.com.

[17] Leskens J., Brugnach M., Hoekstra A., Schuurmans W. (2014). Why are decisions in flood disaster management so poorly supported by information from flood models?. Environ. Model. Softw..

[18] Middelmann-Fernandes M.H. Review of the Australian Flood Studies Database. http://www.ga.gov.au/image_cache/GA15570.pdf.

[19] Wang F., Yuan H. (2010). Challenges of the sensor web for disaster management. Int. J. Digit. Earth.

[20] Bunker D., Levine L., Woody C. (2015). Repertoires of collaboration for common operating pictures of disasters and extreme events. Inf. Syst. Front..

[21] Marufish Disaster Preparedness, Marufish World of Disaster Preparedness. Marufish.com/disaster/flood/.

[22] Fernando M., Gaol F.L. (2018). Designing flood early warning system using internet of things. AIP Conf. Proc..

[23] Bindal A., Kadhim M.H., Parsad D., Patel R.B. (2014). A Pragmatic Review on Algorithmic Approaches for Disaster Management using Wireless Sensor Networks. Int. J. Comput..

[24] Rismayana A.H., Sugianto C.A., Budiyanto I.B. (2018). Prototyping of Flooding Early Warning System using Internet of Things Technology and Social Media. Proceedings of the 3rd Annual Applied Science and Engineering Conference (AASEC 2018).

[25] Ji Z., Anwen Q. The application of internet of things (IOT) in emergency management system in China. Proceedings of the 2010 IEEE International Conference on Technologies for Homeland Security (HST).

[26] Chen T.Y., Wei H.W., Hsu N.I., Shih W.K. A IoT application of safe building in IPv6 network environment. Proceedings of the 2013 IEEE 37th Annual Computer Software and Applications Conference.

[27] Mitra P., Ray R., Chatterjee R., Basu R., Saha P., Raha S., Saha S. Flood forecasting using Internet of things and artificial neural networks. Proceedings of the 2016 IEEE 7th Annual Information Technology, Electronics and Mobile Communication Conference (IEMCON).

[28] Perumal T., Sulaiman M.N., Leong C.Y. Internet of Things (IoT) enabled water monitoring system. Proceedings of the 2015 IEEE 4th Global Conference on Consumer Electronics (GCCE).

[29] Mane S.S., Mokashi M.K. Real-time flash-flood monitoring, alerting and forecasting system using data mining and wireless sensor network. Proceedings of the 2015 International Conference on Communications and Signal Processing (ICCSP).

[30] Du C., Zhu S. (2012). Research on urban public safety emergency management early warning system based on technologies for the internet of things. Procedia Eng..

[31] Kumar S.A., Bao S., Singh V., Hallstrom J. (2019). Flooding disaster resilience information framework for smart and connected communities. J. Reliab. Intell. Environ..

[32] Sarker M.N.I., Peng Y., Yiran C., Shouse R.C. (2010). Disaster resilience through big data: Way to environmental sustainability. Int. J. Disaster Risk Reduct..

[33] Wellington J., Ramesh P. Role of internet of things in disaster management. Proceedings of the International Conference on Innovations in Information Embedded and Communication Systems (ICIIECS).

[34] Rafi M.M., Aziz T., Lodi S.H. (2018). A comparative study of disaster management information systems. Online Inf. Rev..

[35] Shalini E., Surya P., Thirumurugan R., Subbulakshmi S. (2016). Cooperative flood detection using SMS through IoT. Int. J. Adv. Res. Electr. Electron. Instrum. Eng..

[36] Organization of American States Disasters (OAS) (1990). Planning and Development: Managing Natural Hazards to Reduce Loss.

[37] Yao K. (2015). E-Government for Disaster Risk Management.

[38] Eraslan Z.A., Emem O., Helvacı C., Batuk F., Gümüsay U., Demir N., Turk T., Bayram B. (2004). System Design of Desaster Manegment Information System in Turkey as a Part of E-Government.

[39] Ibrahim T. (2020). Conceptual Design of E-governance in Disaster Management System. Ph.D. Thesis.

[40] Lixin Y., Lingling G., Dong Z., Junxue Z., Zhanwu G. (2012). An Analysis on Disasters Management System in China.

[41] Fan C., Mostafavi A. Establishing a framework for disaster management system-of-systems. Proceedings of the Annual IEEE International Systems Conference (SysCon).

[42] Bonnie L., Mindy K., Mary C., Marshall G., Anthony C., Golden G., Sheila S. (2005). Children and Disaster: Age, Gender, and Parental Effects on PTSD Symptoms.

[43] United Nations Office for Disaster Risk Reduction Disaster Risk Management. https://www.undrr.org/terminology/disaster-risk-management.

[44] Asian Disaster Preparedness Center (2016). Primer Series on ICTD for Youth.

[45] Nowell B., Steelman T., Velez A.-L.K., Yang Z. (2018). The structure of effective governance of disaster response networks: Insights from the field. Am. Rev. Public Adm..

[46] Alfahdawi K., Ismaeel H. (2014). Information Technology in Iraq Reality and Challenges.

[47] Cockburn A. (2008). Writing Effective Use Cases: The Agile Software Development Series.

[48] Othman S.H., Beydoun G. (2016). A metamodel-based knowledge sharing system for disaster management. Expert Syst. Appl..

[49] OMG (2003). MDA Guide; USA; Version 1.0.1..

[50] Cook S. (2004). Domain-specific modeling and model driven architecture. MDA J. BPT Column.

[51] Ibrahim T., Mishra A. (2020). Internet of Things (IoT) and Artificial Neural Networks Towards Water Pollution Forecasting. Rocz. Ochr. Sr..

[52] Goniewicz K., Burkle F.M. (2019). Analysis of the Potential of IT System Support in Early Warning Systems: Mitigating Flood Risk in Poland. Prehosp. Disaster Med..

[53] Goniewicz K., Magiera M., Burkle F.M., Goniewicz M. (2020). Prospective Study on the Potential Use of Satellite Data for Disaster Prevention, Preparedness, and Mitigation in Poland.

